# Utero-placental expression of angiotensin-(1–7) and ACE2 in the pregnant guinea-pig

**DOI:** 10.1186/1477-7827-11-5

**Published:** 2013-01-22

**Authors:** Gloria Valdés, Jenny Corthorn, Manish S Bharadwaj, JaNae Joyner, Daniela Schneider, K Bridget Brosnihan

**Affiliations:** 1 Centro de Investigaciones Médicas y Departamento de Nefrología, Escuela de Medicina, Pontificia Universidad Católica, Santiago, Chile; 2 Hypertension and Vascular Research Center, Wake Forest School of Medicine, Winston-Salem, NC, USA

## Abstract

**Background:**

In humans, trophoblast invasion, vascular remodeling and placental development are critical to determine the fate of pregnancy. Since guinea-pigs (GP) and humans share common pregnancy features including extensive trophoblast invasion, transformation of the uterine spiral arteries and a haemomonochorial placenta, the GP animal model was deemed suitable to extend our knowledge on the spatio-temporal immunoreactive expression of the vasodilator arpeptide of the renin-angiotensin system, angiotensin-(1–7) [Ang-(1–7)] and its main generating enzyme, angiotensin converting enzyme 2 (ACE2).

**Methods:**

Utero-placental units were collected in days 15, 20, 40 and 60 of a 64–67 day long pregnancy in 25 Pirbright GP. Ang-(1–7) and ACE2 expression in utero-placental units were evaluated by immunohistochemistry.

**Results:**

Ang-(1–7) and ACE2 were detected in the endothelium and syncytiotrophoblast of the labyrinthine placenta, interlobium, subplacenta, giant cells, syncytial sprouts, syncytial streamers, and myometrium throughout pregnancy. In late pregnancy, perivascular or intramural trophoblasts in spiral and mesometrial arteries expressed both factors. Immunoreactive Ang-(1–7) and ACE2 were present in decidua and in the vascular smooth muscle of spiral, myometrial and mesometrial arteries, which also express kallikrein (Kal), the bradykinin receptor 2 (B2R), vascular endothelial growth factor (VEGF) and its type 2 receptor (KDR), but no endothelial nitric oxide synthase (eNOS). In addition, the signal of Ang-(1–7) and ACE2 was especially remarkable in giant cells, which also show Kal, B2R. eNOS, VEGF and KDR.

**Conclusions:**

The spatio-temporal expression of Ang-(1–7) and ACE2 in GP, similar to that of humans, supports a relevant evolutionary conserved function of Ang-(1–7) and ACE2 in decidualization, trophoblast invasion, vascular remodeling and placental flow regulation, as well as the validity of the GP model to understand the local adaptations of pregnancy. It also integrates Ang-(1–7) to the utero-placental vasodilatory network. However, its antiangiogenic effect may counterbalance the proangiogenic activity of some of the other vasodilator components.

## Background

The outcome of pregnancy depends upon the success of placentation. In normal human pregnancy, extravillous trophoblasts (EVT) after anchoring in the uterine wall migrate through the decidualized stroma to invade the uterine spiral arteries. This last process destroys vascular smooth muscle and replaces the endothelium with EVT by the second trimester of pregnancy. This remodeling increases vessel diameter and creates a high flow, low resistance arteriolar system that meets the increased demands of the fetus across the barrier of the rich *de novo* vasculature of the placenta. The disturbance of this finely tuned orchestrated sequence causes important obstetric and neonatal complications that range from miscarriages due to failed attachment to the extensive invasion of placenta accreta. In the midst, a shallow invasion results in intrauterine growth retardation, or in a hypoperfused placenta which sheds to the maternal circulation microvillous particles and soluble factors that cause the clinical phase of preeclampsia. Among these factors, agonistic autoantibodies to angiotensin II receptor 1 (AT1-AA) participate in the physiopathology of preeclampsia, stressing the prominence of the renin-angiotensin system (RAS) in this condition [1,2].

The RAS is a key regulator of systemic hemodynamics. In normal human pregnancy the vasoconstrictor peptide angiotensin II (Ang II) is elevated [3], but the counterbalancing vasodilator angiotensin-(1–7) [Ang-(1–7)] is increased in plasma and urine [4,5]. Angiotensin-converting enzyme 2 (ACE2), a pleiotropic monocarboxypeptidase [6], is the main Ang-(1–7) generating enzyme and influences the relative expression/functions of Ang II and Ang-(1–7). Ang-(1–7) most likely contributes to the reduction in the systemic vascular resistance and the maintenance of normal systemic blood pressure, as suggested by its defective response in preeclampsia [4] and by the increment of blood pressure in pregnant ACE2 knock-out mice [7]. Apart from vasoactive effects, Ang II stimulates proliferation and angiogenesis [8,9], while Ang-(1–7) is antiproliferative and antiangiogenic [10].

In human utero-placental tissues the traditional vasoconstrictor components of the RAS are ubiquitous [11-16]. As to vasodilator peptides and receptors of the RAS, Broughton-Pipkin and her group have recently shown higher early expression of the receptor of Ang IV in the syncytiotrophoblast and extravillous trophoblast [12]; in addition, our laboratories have shown that Ang-(1–7) and ACE2 are expressed in multiple sites of the utero-placental interface [17,18].

Since vasodilators are key players in the hemodynamic local adaptations of pregnancy, we now aim at understanding the paracrine role of Ang-(1–7) and ACE2 in the utero-placental unit by evaluating the immunohistochemical expression of both factors in the guinea-pig. This caviomorph rodent shares with women: (a) a haemomonochorial placenta; (b) similar progesterone levels and response to progesterone antagonists [19]; (c) an extensive trophoblast invasion and remodeling of the utero-placental arteries [20,21]; (d) the expression of paracrine factors implicated in vasodilation and trophoblast invasion, as matrix metalloproteases (MMPs), kallikrein (Kal), bradykinin 2 receptor (B2R), endothelial nitric oxide synthase (eNOS), vascular endothelial growth factor (VEGF), VEGFR-2 (Flk-1/KDR) and VEGFR-1 (Flt-1) [22,23]; (e) pregnancy toxemia, observed in animals with spontaneous aortic stenosis below the renal arteries or with banded uterine arteries and transected ovarian arteries [24,25]. Accruing further information on the presence and localization of vasodilator components within the RAS in this species will help understand their participation in the local adaptations of human pregnancy.

## Methods

Guinea-pigs (Pirbright white *Cavia porcellus* ~600 g) were housed under controlled humidity, temperature and light cycle conditions. Females were examined daily, and were caged with fertile males when perforation of the vaginal closure membrane ocurred; the day a copulatory plug was observed was defined as day one of pregnancy. Twenty-five dams were sacrificed in early to term pregnancy, on days 15 (n = 4), 20 (n = 6), 40 (n = 7), and 60 (n = 8) of a 64–67 day pregnancy, after being anesthetized with an intraperitoneal injection of ketamine (100 mg/kg) and xylazine (4 mg/kg). The uterus was dissected and feto-placental units (2–5 per dam) were removed; some were sagitally sliced and fixed in a single block, in others the placenta, endometrium, and mesometrium were separated. The animals were euthanized with an overdose of anesthesia. Tissues were fixed immediately with phosphate-buffered 4% formalin for 24 hours.

### Ethical approval

The experiments were conducted according to the *Guide for the Care and Use of Laboratory Animals* (National Research Council, USA) and approved by the Ethics Committee of FONDECYT (Fondo Nacional de Desarrollo Científico y Tecnológico, Chile).

### Immunohistochemistry

Fixed tissues were paraffin embedded, sectioned (5 μm), dehydrated in a graded series of ethanol and xylene and included in Paraplast-Plus® (Sigma, St. Louis, MO). Sections were deparaffinized in xylene and rehydrated in a graded alcohol series. Endogenous peroxidases were blocked with incubation in 10% H_2_O_2_ for 10 minutes. Sections were incubated in a humid chamber for 30 minutes with goat serum (Vector Laboratories, Burlingame, CA) as a protein block followed by incubation for 18 hours at 4°C with the primary antibodies. These antibodies were affinity-purified polyclonal rabbit anti-Ang-(1–7) (1:25) and anti-ACE2 (1:1000) produced by our group at Wake Forest School of Medicine [18,26]. Sections were immunostained using a biotin-streptavidin-peroxidase system (LSAB+®, DakoCytomation, Carpinteria, CA). Finally, the samples were treated for 15 minutes with 0.1% (w/v) 3-3'-diaminobenzidine (Sigma) in buffer containing 0.05% H_2_O_2_. The slides were counterstained with Harris hematoxylin (Sigma).

Cytotrophoblasts were identified by staining with an antipancytokeratin mouse monoclonal antibody (1:100, P2871, Sigma). Smooth muscle and endothelial cells were characterized with antibodies against α-smooth muscle actin (1A4, 1:1500) and von Willebrand factor (A0082, 1:400) respectively, both from DakoCytomation, Carpinteria, CA. The specificity of the staining was determined by eliminating the primary antibody, incubating with non-specific immunoglobulin or by preadsorbing with the peptide to which the antibody was generated.

Previous studies which were revised in the context of the present work [22,23] were performed with the following antibodies: rabbit polyclonal antiserum against purified rat urinary kallikrein (1:2000), mouse monoclonal anti-eNOS (1:50, BD Transduction Laboratories, Lexington, KY), VEGF (1:50, Upstate Laboratories, Syracuse, NY) and rabbit polyclonal KDR (1:500, Upstate). The B2R antibody (1:4000), kindly donated by Dr. Werner Müller-Esterl, Frankfurt Universitäts, Germany, was raised against the rat kinin B2R and recognizes the various domains of the receptor.

## Results

**Figure 1:**
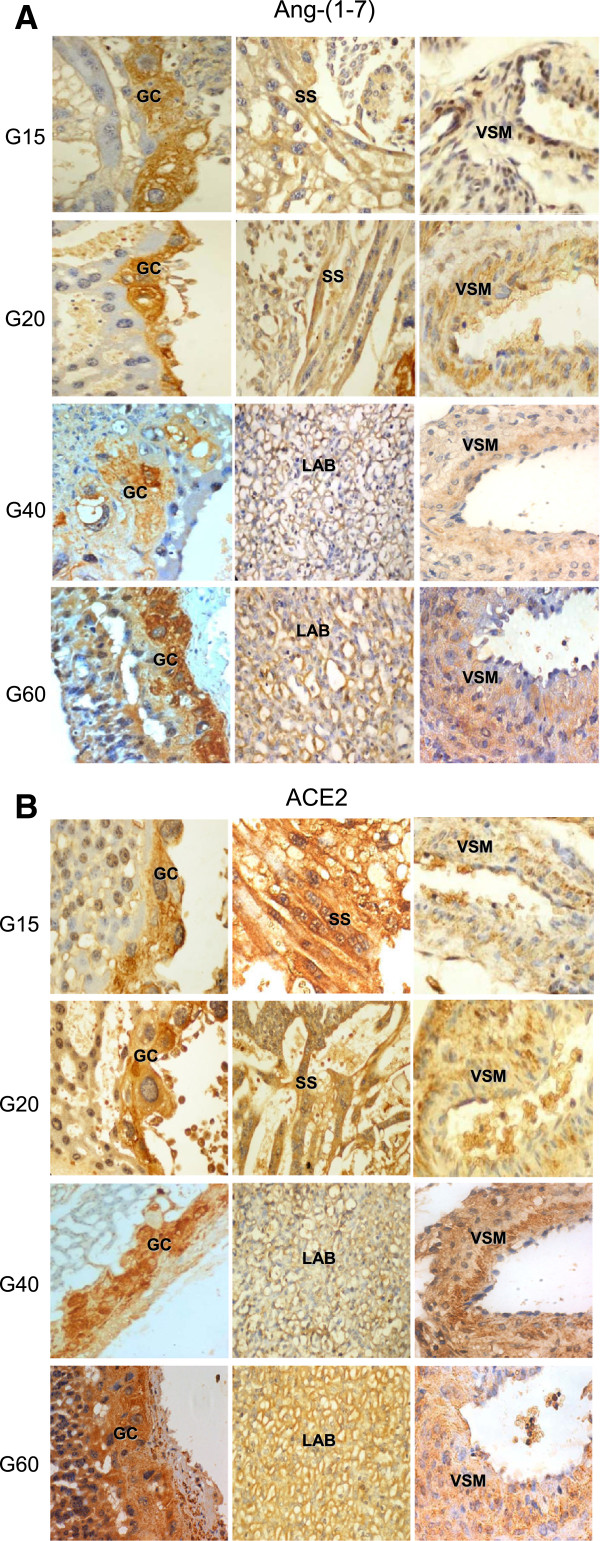
**Ang-(1–7) immunoreactivity in different stages and structures of the utero-placental unit.** This expression (orange-brown) is depicted in giant cells (GC), syncytial streamers (S), labyrinth (LAB) and vascular smooth muscle (VSM) in myometrial artery of gestational days (G) 14, 20, 40 and 60 of the pregnant guinea-pig. **(B) ACE2 immunoreactivity in different stages and structures of the utero-placental unit.** This expression (orange-brown) is depicted in giant cells, syncytial streamers and vascular smooth muscle of myometrial artery of gestational days (G) 15, 20, 40 and 60 of the pregnant guinea-pig. (x400).

In embryonic derived cells immunoreactivity for Ang-(1–7) (Figure 1A) and ACE2 (Figure 1B) was detected in the interlobium, endothelium and syncytiotrophoblast of the labyrinthine placenta, subplacenta, giant cells, syncytial sprouts, syncytial streamers and intravascular and intraluminal trophoblasts (Table 1).

**Table 1:** Distribution of Angiotensin-(1–7) and ACE2 in the guinea-pig utero-placental unit in early (day 15), mid (days 20 and 40) and late (day 60) gestation

**Day of Gestation**	**Giant cells**	**Interlobium**	**Labyrinth**	**Subplacenta**	**Decidual cells**	**Streamers**	**Spiral arteries**	**SM spiral arteries**	**Myometrium**	**Mesometrium**
***Ang-(1–7)***										
*Day 15*	+	+	Not recognizable	±	+	+	VSM	+	+	VSM
*Day 20*		+	+	+	+	+	VSM	+	+	VSM
*Day 40*	+	SCT, endothelium	SCT, endothelium	+	+	+	VSM	+	+	VSM
*Day 60*	+	+	+	Necrotic	Necrotic	Necrotic	VSM, IVTB	+	+	VSM, endothelium
***ACE2***										
*Day 15*	+	+	Not recognizable	±	+	+	VSM	+	+	VSM
*Day 20*	+	+	+	+	+	+	VSM	+	+	VSM
*Day 40*	+	SCT, endothelium	SCT, endothelium	+	±	±	VSM	+	+	VSM
*Day 60*	+	SCT, endothelium	SCT, endothelium	Necrotic	Necrotic	Necrotic	VSM, IVTB	VSM, IVTB	+	VSM, endothelium

SCT = syncytiotrophoblast; IVTB =intravascular trophoblast; VSM = smooth muscle. Necrotic areas in late gestation in the central portion of the utero-placental unit were identified by the disruption of the tissues. Negative and positive sections observed in different animals, or faint signal (±); positive signal in all animals (+).

**Figure 2:**
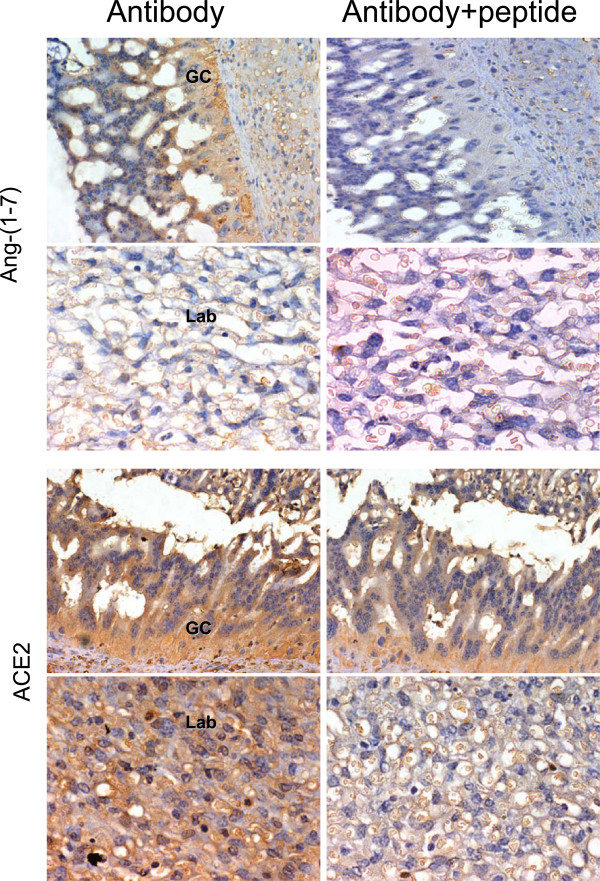
**Immunoreactive expression of Ang-(1–7) and ACE2 in giant cells (GC) and labyrinth (LAB), from a gestational day 60 pregnant guinea-pig.** Sections were preincubated with and without the immunizing peptide. (x400).

In maternal tissues, decidua, vascular smooth muscle and endothelial cells of spiral and mesometrial arteries, as well as the myometrium, showed Ang-(1–7) and ACE2 (Figures 1 and 2, Table 1). The decidual expression of ACE2 was stronger in gestational days 15 and 20 as compared to day 40. In late gestation (day 60), the necrotic areas in the subplacenta, decidual cells, and streamers hindered the observation of the expression of Ang-(1–7) and ACE2 in these zones.

The signal for both Ang-(1–7) and ACE2 was especially remarkable in the markedly protrusive parietal giant cells that envelop the maternal blood sinuses with long cytoplasmic laminas (Figures 1 and 2).

Ang-(1–7) and ACE2 expression was coarsely granular in giant cells and vascular smooth muscle, finely granular in the labyrinth, interlobium and decidual cells, and diffuse with a peripheral reinforcement in syncytial streamers.

Temporal assessment showed that Ang-(1–7) and ACE2 were expressed throughout pregnancy in the giant cells, labyrinth and myometrial arteries. As early as day 15 of gestation, invasive trophoblasts coalesced into synctyial streamers and migrated into the endometrium; their immunoreactive expression of Ang-(1–7) and ACE2 was more intense in early and mid pregnancy as compared to late pregnancy, when streamers were diminished. In the myometrium, spiral arteries were surrounded by Ang-(1–7) and ACE2 positive trophoblasts. In days 40 and 60 of late gestation, uterine and mesometrial arteries were multilayered, and displayed Ang-(1–7) and ACE2 in vascular smooth muscle and invading trophoblasts.

Control sections incubated with non-specific immunoglobulin or in the absence of the first antibody yielded no staining in different structures and stages of pregnancy (not shown). Sections preincubated with an excess of peptide that corresponds to the epitope recognized by the antibody showed no immunoreactive signal for Ang-(1–7), while for ACE2 some background staining persisted (Figure 2).

## Discussion

The spatio-temporal expression of Ang-(1–7) and ACE2 in sites related to decidualization, trophoblast invasion, vascular remodeling, placental flow regulation and platelet antiaggregation supports a relevant role of these peptides/proteins in the gestation of the guinea-pig.

The coincident location of Ang-(1–7) and ACE2 in the guinea-pig and in equivalent human structures as reported by Pringle et al. [11] and by us [18] endorses an evolutionary conserved role, and provides further confirmation of the validity of the guinea-pig model to understand human pregnancy. The shift of the predominance of Ang II in the uterine fundus to Ang-(1–7) in the placental bed [18] and the increased expression of Ang-(1–7) and ACE2 mRNA in early pregnancy as compared to normal term pregnancy [17 are indicative of an early vasodilatory effect on the maternal vasculature.

Rats, as guinea-pigs, express Ang-(1–7) and ACE2 staining in the labyrinthine placenta [26]. The marked decrement of placental and uterine Ang-(1–7) concentrations in the reduced uterine perfusion pressure rat model of hypertensive pregnancy [26] supports a role of this vasodilator in normotensive pregnancy.

**Figure 3:**
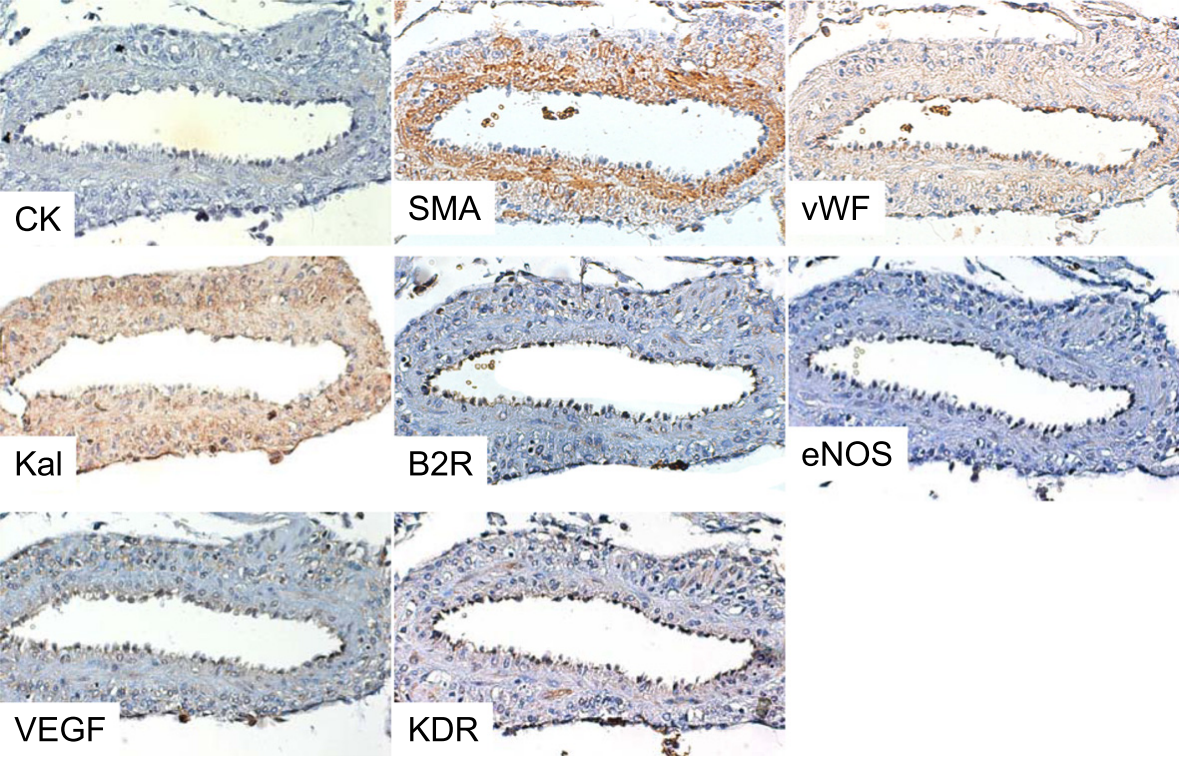
**Sequential sections of the myometrial artery in gestational day 40, which in Figure**1A** and B displays immunoreactivity for Ang-(1–7) and ACE2.** These show a moderate signal for kallikrein (Kal), a faint and scanty one for bradykinin R2 (B2R), vascular endothelial growth factor (VEGF) and its type 2 receptor (KDR), and no endothelial nitric oxide synthase (eNOS), in vascular smooth muscle (VSM) characterized by α-smooth muscle actin. These vasodilators are present in endothelial cells characterized by von Willebrand factor (vWF). Absence of cytokeratin (CK) positive cells demonstrates no intraarterial extravillous trophoblasts. (x200).

The localization of Ang-(1–7) in the same cell types that express other vasodilator factors in this species (Kal, the B2R, eNOS, VEGF and KDR) [22,23] highlights the importance and complexity of the utero-placental vasodilator network [27]. As in women, Ang-(1–7) and ACE2 in the guinea pig are additionally expressed in decidual cells. The vascular smooth muscle of myometrial and mesometrial arteries display intense immunoreactivity for Ang-(1–7) and ACE2, a moderate signal for kallikrein, a faint signal in scant cells for B2R, VEGF and KDR and absence of eNOS (Figure 3). This localization of the paracrine/autocrine vasodilator components of the RAS in both maternal and fetal components of the utero-placental unit, suggests their instrumental participation in trophoblast invasion and spiral artery remodeling. The preparation of arteries for trophoblast invasion has been mainly attributed to the nearby trophoblasts, uterine natural killer cells and macrophages. In this case, it is feasible that Ang-(1–7) generated by the vascular smooth muscle interacts with NO liberated by its “pacemaker”, the approaching trophoblast [28]. The relevance of uterine artery vasodilatation in pregnancy has been underscored in the ewe, by the demonstration that even vasoconstrictor Ang II induces in them PGI_2_ and NO endothelial production [29].

**Figure 4:**
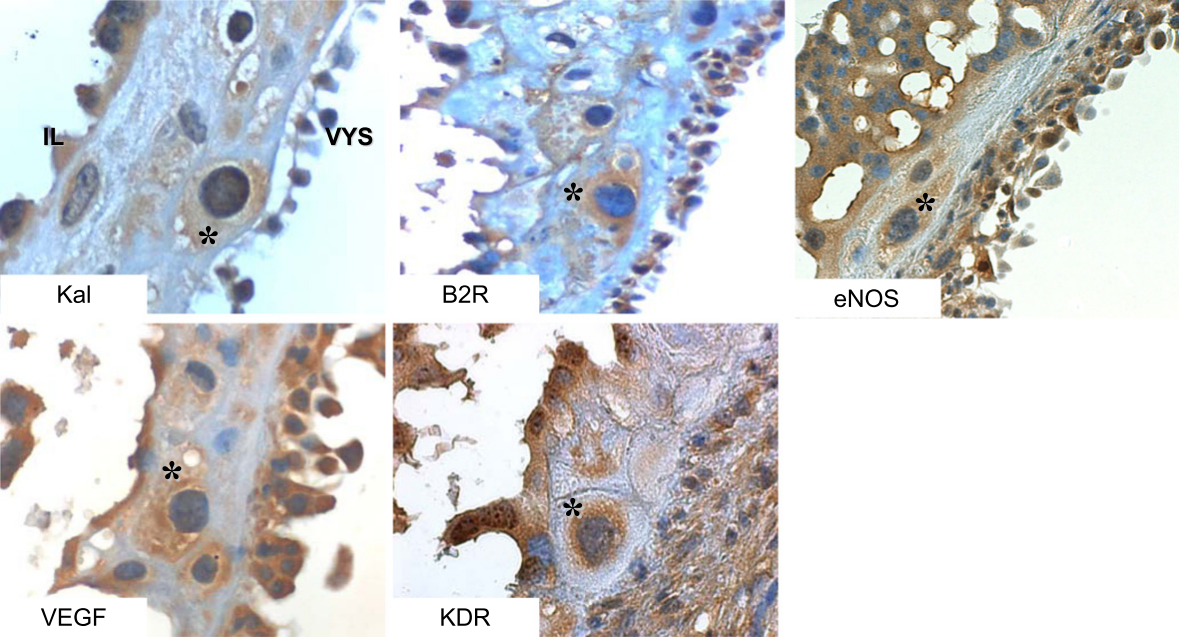
**Giant cell (GC) expression of vasodilator factors.** The signal is intense for vascular endothelial growth factor (VEGF), moderate for its type 2 receptor (KDR), kallikrein (Kal), the bradykinin B2 receptor (B2R) and endothelial nitric oxide synthase (eNOS) in two different sets of sequential sections (upper and lower panels). Asterisks indicate the same cell in each panel. Visceral yolk sac=VYS, interlobium=IL. (x400).

The intense expression of Ang-(1–7) and ACE2 in giant cells observed in this study made us re-assess in them our previous analysis on the immunoreactive expression of the vasodilator panel in the guinea pig [22,23]. In sequential sections we found a strong immunoreactive signal for VEGF and a moderate signal for kallikrein, the B2R, eNOS, and KDR (Figure 4). In future studies the prominence of Ang-(1–7) and ACE in parietal giant cells needs to be examined in regard to their functional importance in decidualization, trophoblast invasion, synthesis of a wide range of hormones, angiogenic and vasodilatory factors (VEGF, NO) and glycogen and glycoproteins storage [28,30]. If their hormonal production is disturbed, vascularization of the decidua is compromised, and embryos present lethal defects. In mice, inhibition or overabundance of giant cells are equally detrimental.

In contrast to VEGF, NO, bradykinin and Ang II, pleitropic Ang-(1–7) is a potent antiagiogenic factor, which impairs tube formation by human umbilical cells [31], growth of tumors and tumoral cells and vascularization of sponge implants [32-34]. The importance of ACE2 in mediating the Ang II-Ang-(1–7) balance is supported by growth of pancreatic cancer cells when its mRNA is silenced and by inhibition of tumor expansion when the enzyme is overexpressed [35].

The localization of Ang-(1–7) and ACE2 in different structures of the guinea-pig utero-placental unit tallies with a role of these factors in the regulation of trophoblast invasion, angiogenesis and blood flow. The findings described herein show that the guinea-pig represents an attractive model to manipulate the opposing arms of the RAS in order to understand the functionality of the system in the systemic and local adaptations of pregnancy. Adding a new species to the mice and rat models now in use will especially contribute to the study of the placental bed along pregnancy, since this crucial territory is out-of-bounds for research in ongoing human pregnancies.

## Conclusions

The spatio-temporal expression of Ang-(1–7) and ACE2 in GP, similar to that of humans, supports a relevant evolutionary conserved function of Ang-(1–7) and ACE2 in decidualization, trophoblast invasion, vascular remodeling and placental flow regulation, as well as the validity of the GP model to understand the local adaptations of pregnancy. It also integrates Ang-(1–7) to the utero-placental vasodilatory network. However, its antiangiogenic effect may counterbalance the proangiogenic activity of some of the other vasodilatory components.

## Competing interests

The authors declare that they have no competing interests.

## Authors’ contributions

The study was conceived by GV and KBB; the design, the histological analysis, the interpretation of the data and the drafting of the manuscript were done by GV and JC; JC sacrificed the animals and prepared the tissue sections; she and DS performed the immunohistochemistry of non-RAS factors; MSB and JJ performed the inmmunohistochemistry for Ang-(1–7) and ACE2; KBB participated in the analysis of the data and in the different versions of the manuscript. All authors read and approved the final manuscript.
